# Prediction of fracture risk in men: A cohort study

**DOI:** 10.1002/jbmr.1498

**Published:** 2011-12-20

**Authors:** Liisa Byberg, Rolf Gedeborg, Thomas Cars, Johan Sundström, Lars Berglund, Lena Kilander, Håkan Melhus, Karl Michaëlsson

**Affiliations:** 1 Department of Surgical Sciences, Orthopedics, Uppsala UniversityUppsala, Sweden; 2 Department of Surgical Sciences, Anesthesiology, Intensive Care Medicine, and Pain Treatment, Uppsala UniversityUppsala, Sweden; 3 Department of Public Health and Caring Sciences, Geriatrics, Uppsala UniversityUppsala, Sweden; 4 Department of Medical Sciences, Acute and Internal Medicine, Uppsala UniversityUppsala, Sweden; 5 Uppsala Clinical Research Centre, Uppsala UniversityUppsala, Sweden; 6 Department of Medical Sciences, Osteoporosis and Clinical Pharmacogenetics, Uppsala UniversityUppsala, Sweden

**Keywords:** COMORBIDITY, LIFESTYLE, MEDICINE, FRACTURE, PREDICTION

## Abstract

FRAX is a tool that identifies individuals with high fracture risk who will benefit from pharmacological treatment of osteoporosis. However, a majority of fractures among elderly occur in people without osteoporosis and most occur after a fall. Our aim was to accurately identify men with a high future risk of fracture, independent of cause. In the population-based Uppsala Longitudinal Study of Adult Men (ULSAM) and using survival analysis we studied different models' prognostic values (R^2^) for any fracture and hip fracture within 10 years from age 50 (*n* = 2322), 60 (*n* = 1852), 71 (*n* = 1221), and 82 (*n* = 526) years. During the total follow-up period from age 50 years, 897 fractures occurred in 585 individuals. Of these, 281 were hip fractures occurring in 189 individuals. The rates of any fracture were 5.7/1000 person-years at risk from age 50 years and 25.9/1000 person-years at risk from age 82 years. Corresponding hip fractures rates were 2.9 and 11.7/1000 person-years at risk. The FRAX model included all variables in FRAX except bone mineral density. The full model combining FRAX variables, comorbidity, medications, and behavioral factors explained 25% to 45% of all fractures and 80% to 92% of hip fractures, depending on age. The corresponding prognostic values of the FRAX model were 7% to 17% for all fractures and 41% to 60% for hip fractures. Net reclassification improvement (NRI) comparing the full model with the FRAX model ranged between 40% and 53% for any fracture and between 40% and 87% for hip fracture. Within the highest quintile of predicted fracture risk with the full model, one-third of the men will have a fracture within 10 years after age 71 years and two-thirds after age 82 years. We conclude that the addition of comorbidity, medication, and behavioral factors to the clinical components of FRAX can substantially improve the ability to identify men at high risk of fracture, especially hip fracture. © 2012 American Society for Bone and Mineral Research.

## Introduction

Osteoporotic fractures, especially hip fractures, constitute a large problem for the elderly population and, in terms of health care costs, for society.^1^, ^2^ Therefore, preventive measures to reduce the number of fractures are of great importance. To do this, people at high risk for fracture need to be identified. Several fracture risk scoring tools have been presented.^3^ The most widely used, the FRAX algorithm, was designed to identify high fracture risk individuals likely to benefit from pharmacologic treatment to increase bone mineral density (BMD)^4^–^6^ and thereby to reduce their fracture risk.^1^ However, more than 80% of low-trauma fractures occur in people who do not have osteoporosis,^7^ implying that they may not benefit from pharmacological treatment. The risk of fracture is affected by the risk for falls and by bone architecture. These two main determinants are in turn influenced by environmental factors, age, genes, lifestyle behaviors, diseases, and medications.^8^–^11^ Thus, in some individuals, prevention of falls can reduce the risk of fractures,^11^–^15^ sometimes in combination with treatment for low BMD.

A majority of patients with hip fracture present with comorbidities when admitted to the hospital^16^ but it is not known how much of the variation in fracture risk comorbid conditions and medications can explain. Lifestyle and social factors not included in FRAX have also been associated with fracture risk.^17^, ^18^ FRAX calculates the predicted individual absolute 10-year risk of osteoporotic fracture, based on 11 clinical risk factors and, optionally, BMD.^19^ Although validated in several cohorts,^5^, ^20^ it is not known how well the FRAX variables perform in comparison with these factors or whether the potential differences in performance change with increasing age.

In the present study, we investigate to what extent variables included in FRAX, comorbidities, medications, behavioral factors, and a combination of these four components can explain the variation in fracture risk at different ages in a population-based cohort of 50-year-old men followed with repeat examinations for 40 years.

## Subjects and Methods

**Figure 1:**
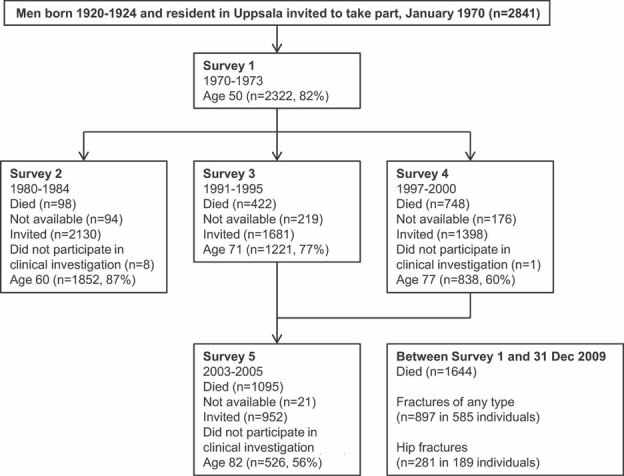
Flow chart describing the present study. Deaths are presented as cumulative mortality from start of survey 1. Numbers not available represent those who were not living in the Uppsala region at time of invitation. They did not contribute risk factor information at that survey but they could return for a later survey if they had moved back to Uppsala. All men were traced in patient registers for fracture data, including those “not available.” Men not participating in the clinical investigation only completed questionnaires and were not included in our analysis.

The Uppsala Longitudinal Study of Adult Men (ULSAM) has been described previously^21^ and is outlined in Figure 1. All men born from 1920 to 1924 and living in Uppsala municipality (*n* = 2841), Sweden, in January 1970 were invited to a health investigation in which 2322 men aged around 50 years participated. The men were re-investigated at 60 (*n* = 1852), 71 (*n* = 1221), 77 (*n* = 838), and 82 (*n* = 526) years of age. Information was collected by clinical investigation and by questionnaires at each survey.^21^ The Uppsala University Ethics Committee approved the study and all participants gave their informed consent before taking part in the study.

We primarily used four predefined categories of exposure variables: FRAX variables (V_FRAX_), comorbidities, medications, and behavioral factors.

### V_FRAX_

Our model, V_FRAX_, included the following components of FRAX^19^: age, height and weight (continuous), previous fracture (yes, no), parent hip fracture (age 71 years: yes, no), current smoking (yes, no), glucocorticoid use (yes, no), rheumatoid arthritis (yes, no), alcohol use (high versus lower amounts), and secondary osteoporosis (yes, no). Secondary osteoporosis was categorized as “yes” based on the presence of liver disease, type 1 diabetes mellitus, hypogonadism, malnutrition, or thyreotoxicosis (Supporting Table 1).^22^ We included the separate variables without interaction terms in V_FRAX_ because the beta coefficients for the variables in FRAX are not published. Because all interactions in FRAX, however, are dependent on age^23^ and the men in our cohort had a similar age, the impact on our estimates of not considering the interaction terms is probably modest.

### Comorbidity

Information on comorbidity at each investigation was extracted from the National Patient Register (NPR) using the unique personal identification number given to all Swedish citizens. We used information from primary diagnosis as well as information from up to five secondary diagnoses. We used a modified and expanded comorbidity score based on Elixhauser's comorbidity score,^24^ with adaption to the Swedish versions of the International Classification of Diseases (ICD, 10th edition [ICD-10]; KSH97), ICD-9 (ICD, 9th edition; KSH87), and ICD-8 (ICD, 8th edition)^25^ (Supporting Table 1). The 39 comorbidity items were further collapsed into three major disease groups: cardiovascular diseases, cancer, and other diseases. Diabetes mellitus type 2 was diagnosed at the clinical investigations.

### Medications

Medications reported by the participant at the time of each investigation were grouped according to major categories of the Anatomical Therapeutic Chemical (ATC) classification system (Supporting Table 2).^26^

### Behavioral characteristics

In addition to smoking habits (never, former, current) and alcohol consumption (described three paragraphs above), we included physical activity (low, moderate, high), educational level (age 50 years, at least high school: yes, no), and whether the person lived alone (ages 71, 77, and 82 years: yes or no). Information on marital status and physical activity at work estimated from occupational groups^18^ were retrieved from the Swedish censuses from 1960, 1970, and 1980. The functional risk factor cognitive impairment,^13^ defined as previously described,^27^ was based on cognitive function tests performed at ages 71, 77, and 82 years.^28^

### Additional exposure information

In samples from the age 71 years and age 82 years investigations, plasma 25-hydroxyvitamin D concentrations were determined using high-pressure liquid chromatography–mass spectrometry.^29^ To take into account seasonal variation in vitamin D, we categorized the season of blood draw as summer (May–October) and winter (November–April).^29^ Concentrations of serum retinol were determined in samples from ages 50, 70, and 82 years, using high-performance liquid chromatography.^30^

For the age 82 years investigation, BMD (g/cm^2^) of the femoral neck was measured using dual-energy X-ray absorptiometry (DXA) (Lunar Prodigy, Lunar Corp., Madison, WI, USA; *n* = 461).^31^ Both extremities were used in the calculation when applicable.

We calculated the cumulative number of falls that occurred before each investigation based on information in the NPR (Supporting Table 1). In addition, at age 71 years, the number of self-reported falls during the previous year was reported as none, 1 to 2 times, and 3 times or more.

### Outcomes

Our main outcomes, any fracture (ICD-10 codes: S12, S22, S32, S42, S52, S62, S72, S82, or S92) and hip fracture (ICD-10 codes: S720, S721, or S722), were retrieved from the NPR and outpatient-treated fractures were collected from outpatient registers.^18^ Incident fracture admissions were separated from readmissions of a previous fracture using a previously validated and accurate method.^32^ We studied incident fractures after each age of investigation; ie, 50, 60, 71, 77, and 82 years. Previous fractures were recorded. Time to the second of two fractures occurring within 10 years from each age was considered as secondary outcome.

We chose not to present the results from the age 77 years survey because the follow-up time was covered by the 10-year follow-up periods from ages 71 and 82 years.

### Statistical analyses

Statistical analyses were performed using Stata 11.0 (Stata Corp., College Station, TX, USA). Kaplan-Meier failure curves are presented for all fractures, hip fractures, and two fractures. Cumulative incidence curves for each outcome taking competing risk from mortality into account only marginally deviated from the Kaplan-Meier curves. Using Cox proportional hazards regression we primarily studied five different models—V_FRAX_, comorbidity model, medication model, behavioral factors model, and full model—using the different survey ages as baseline with 10 years of follow-up and with follow-up until end of study (December 31, 2009) with censoring at time of event, time of emigration, or time of death, whichever occurred first and before end of follow-up. Thus, we derived new models for each ∼10-year interval and outcome to take into account changes in exposure prevalences and parameter estimates. Although all men had a similar age, age was nevertheless added as a linear term in each model to take into account the limited variation in age. We additionally studied nested models by combining V_FRAX_ with comorbidity, medication, and behavioral characteristics to investigate whether each component contributed to the R^2^ value. The hazards of our models were considered proportional, indicated by testing using Shoenfeld's residuals and by investigating Nelson-Aalen graphs.

Models were compared with the prognostic value given by Royston's R^2^, a measure of how much of the variation in time to event that can be explained by a model.^33^ Taking the number of covariates in the models into account^33^ did not change the interpretation of our results (data not shown).

The incremental discriminative ability of the full model compared with V_FRAX_ was explored using several methods. The category-free net reclassification improvement (NRI) was calculated using improved predicted survival calculated based on a Weibull model.^34^ Wald and Morris' Risk-Screening Converter^35^ was used for calculation of sensitivity (for a false-positive rate of 10%) and odds of being affected given a positive result (OAPR) using the two models. For both models we compared the highest quintile of predicted hazard with the lowest quintile using Cox regression. The OAPR is interpreted as the ratio of the number of men with fracture to those who did not fracture within the highest quintile. The relative integrated discrimination improvement (rIDI)^36^, ^37^ was based on logistic regression. IDI and the category-free NRI are suggested to be more sensitive estimates of discrimination and reclassification than the previously commonly used C statistic.^34^, ^36^ As a comparison, we calculated Harrell's C for both models using the “estat concordance” command in Stata. Moreover, we assessed the added discriminatory benefit of plasma vitamin D, serum retinol, BMD, and history of falls using rIDI.

To avoid inflated estimates,^38^ we report R^2^, NRI, and Harrell's C as median and 95% confidence interval (CI) derived from 1000 bootstrap samples drawn.

Missing data were imputed using one of the following methods in hierarchical order: (1) the last observation carried forward; (2) the next observation carried backward; and (3) the median value.^39^ For example, physical activity was the most common missing variable at age 60 years (*n* = 144, of which 130 were replaced with information from age 50 years, 10 with information from age 70 years, and four with the median value at age 60 years) and the second most common was height (*n* = 14, all replaced by information from age 50 years). Sensitivity analysis with restriction to nonmissing data did not change the interpretation of our results (data not shown).

## Results

Subjects' characteristics at the different baseline ages are presented in Table 1. The number of comorbidities and medications used increased and the proportion of current smokers decreased with increasing age.

**Table 1:** Characteristics of the Study Subjects at the Different Baseline Ages

	50 years	60 years	71 years	82 years
*n*	2322	1852	1221	526
Age, year, mean (SD)	49.6 (0.6)	59.8 (1.8)	71.0 (0.6)	81.7 (0.9)
FRAX variables				
Height, cm, mean (SD)	176 (6)	176 (6)	175 (6)	173 (6)
Weight, kg, mean (SD)	77.8 (11.1)	78.7 (11.4)	80.3 (11.5)	78.1 (11.2)
BMI, kg/m^2^, mean (SD)	25.0 (3.2)	25.5 (3.3)	26.3 (3.4)	26.1 (3.4)
Previous fracture	26 (1)	110 (6)	138 (11)	104 (20)
Parent fractured hip^a^	109 (5)	106 (6)	109 (9)	47 (9)
Current smoker	1185 (51)	584 (32)	173 (14)	31 (6)
Glucocorticoids	7 (0.3)	17 (0.9)	119 (10)	40 (8)
Rheumatoid arthritis	0 (0)	0 (0)	3 (0.2)	4 (0.8)
Secondary osteoporosis^b^	19 (0.8)	21 (1)	19 (2)	11 (2)
High alcohol consumption	222 (10)	222 (12)	134 (11)	52 (10)
Medications				
None	2100 (90)	1163 (63)	404 (33)	87 (16)
Comorbidities				
None	2149 (92)	1192 (64)	478 (39)	108 (20)
1 comorbidity	142 (6)	411 (22)	355 (29)	109 (21)
2 comorbidities	23 (1)	146 (8)	199 (16)	92 (17)
≥3 comorbidities	8 (0.3)	103 (5)	189 (15)	217 (41)
Cardiovascular disease	35 (2)	217 (12)	316 (26)	263 (50)
Cancer	6 (0.3)	33 (2)	75 (6)	85 (16)
Other diseases	140 (6)	532 (29)	589 (48)	352 (67)
Behavioral factors				
Former smoker	552 (24)	721 (39)	574 (47)	52 (10)
Low leisure-time physical activity level	337 (14)	221 (12)	61 (5)	79 (15)
Moderate leisure-time physical activity level	884 (38)	941 (51)	437 (36)	189 (36)
High leisure-time physical activity level	1101 (47)	690 (37)	723 (59)	258 (49)
Sedentary work	266 (11)	234 (13)	Not assessed	Not assessed
Physically demanding work	253 (11)	160 (9)	Not assessed	Not assessed
Married	1999 (86)	1541 (83)	985 (81)	381 (72)
Living alone	Not assessed	Not assessed	192 (16)	137 (26)
Educational level less than high school	1931 (83)	1556 (84)	1002 (82)	406 (77)
Impaired cognitive function	Not assessed	Not assessed	140 (12)	173 (33)

Data are given as number (percentage) of men unless otherwise stated.

a Assessed at age 71 years.

b Secondary osteoporosis includes: liver disease, type 1 diabetes mellitus, malnutrition, thyreotoxicosis, hypogonadism.

**Figure 2:**
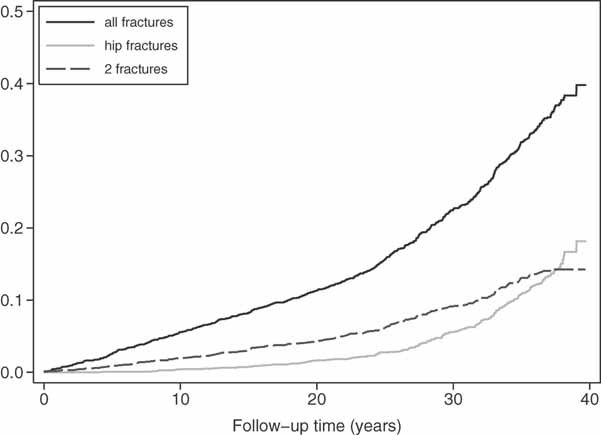
Fracture incidence in 50-year-old men. Kaplan-Meier failure estimates for a first fracture of any type, a first hip fracture, and first two consecutive fractures from age 50 years in Swedish men in the Uppsala Longitudinal Study of Adult Men (ULSAM).

During the total follow-up period (median: 32 years, maximum: 40 years), 897 fractures occurred in 585 individuals (25%). Of these, 281 (31%) were hip fractures occurring in 189 individuals. Figure 2 displays the distribution of the fracture events by years after cohort entry.

**Figure 3:**
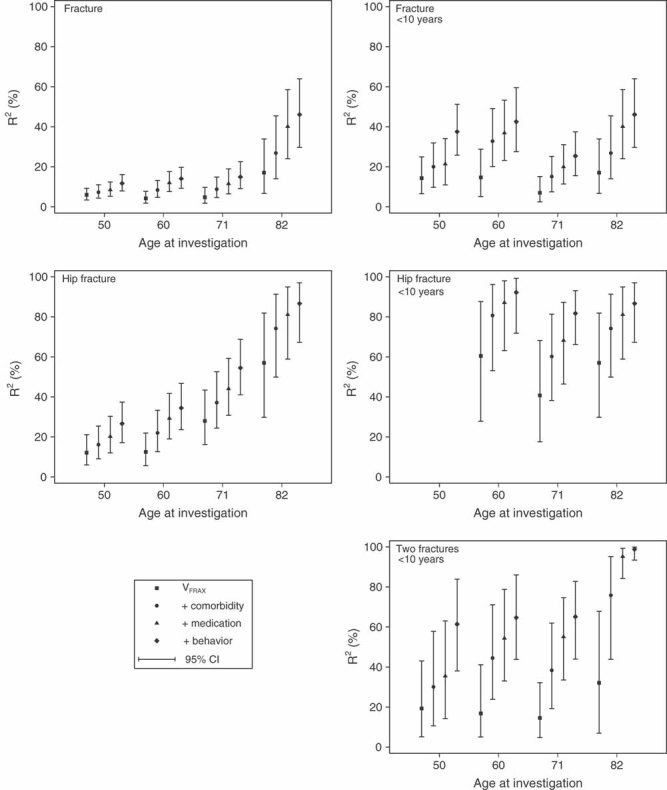
Nested models explained variation in fracture risk. Explained variation (R^2^, 95% confidence intervals) of risk of any fracture, two fractures, and hip fracture, for nested models at different ages and for different follow-up times.

The prognostic values of the four different sets of risk factors and their combination are presented in Table 2. The full model combining V_FRAX_, comorbidity, medications, and behavioral factors had the highest prognostic value for all outcomes and all ages. Investigating models in which one model is added to the previous model showed that each model contributed to an improved prediction of fracture at all ages (Fig. 3). All models had a higher R^2^ value for fractures occurring within 10 years from each baseline than for the whole follow-up period. We focus on the 10-year results in the following paragraphs.

**Table 2:** Variation in Fracture Rate Explained by Different Models (R^2^, %) and rIDI

				R^2^ (95% confidence interval), %					
					
	Fractures, *n* (%)	PYAR	Rate/1000 PYAR	V_FRAX_^b^	Comorbidity model^c^	Medication model^d^	Behavioral model^e^	Full model^f^	rIDI^a^, % (*p*)
Any fracture during follow-up									
From 50 years	585 (25)	61167.4	9.6	6.0 (3.5–9.3)	1.8 (0.6–3.7)	1.2 (0.3–2.9)	6.9 (4.2–10.6)	11.7 (8.0–16.1)	44.5 (<0.001)
From 60 years	416 (22)	34830.6	11.9	4.3 (1.8–7.8)	4.3 (1.8–7.6)	4.6 (1.9–8.4)	4.5 (2.3–7.6)	14.1 (9.3–19.7)	177.1 (<0.001)
From 71 years	254 (21)	14297.6	17.8	4.8 (1.8–9.7)	3.8 (1.3–7.8)	4.0 (1.1–8.6)	5.2 (2.0–10.7)	14.9 (9.2–22.6)	135.0 (<0.001)
From 82 years^g^	64 (12)	2473.5	25.9	17.1 (6.7–33.9)	9.9 (3.3–22.8)	18.9 (7.0–35.4)	14.1 (5.1–30.0)	46.1 (29.7–64.0)	235.5 (<0.001)
Any fracture within 10 years									
From 50 years	126 (5)	22075.7	5.7	14.3 (6.5–24.9)	6.1 (1.6–14.2)	1.7 (0.2–6.7)	28.6 (17.7–42.5)	37.5 (25.8–51.3)	242.6 (<0.001)
From 60 years	109 (6)	16933.2	6.4	14.7 (5.2–28.8)	18.5 (8.3–33.2)	9.9 (2.9–20.8)	11.8 (4.9–21.6)	42.5 (27.6–59.5)	210.0 (<0.001)
From 71 years	143 (12)	10360.3	13.8	7.0 (2.5–15.2)	7.1 (2.4–14.4)	6.2 (1.7–14.2)	8.4 (3.2–16.7)	25.4 (15.6–37.5)	230.5 (<0.001)
From 82 years^g^	64 (12)	2473.5	25.9	17.1 (6.7–33.9)	9.9 (3.3–22.8)	18.9 (7.0–35.4)	14.1 (5.1–30.0)	46.1 (29.7–64.0)	235.5 (<0.001)
Two fractures within 10 years									
From 50 years	29 (1)	22553.3	1.3	19.3 (5.1–43.0)	11.7 (2.5–33.9)	5.5 (0.5–18.2)	44.2 (24.6–67.9)	61.4 (38.1–83.9)	358.7 (0.002)
From 60 years	24 (1)	17318.0	1.4	16.8 (5.1–41.1)	28.7 (10.9–51.3)	16.6 (6.1–35.5)	29.1 (11.3–54.1)	64.7 (43.8–86.0)	214.8 (0.020)
From 71 years	39 (3)	10780.1	3.6	14.6 (4.7–32.2)	22.8 (7.8–44.4)	23.5 (9.6–43.5)	22.5 (8.8–43.3)	65.1 (44.0–82.7)	1440 (0.003)
From 82 years^h^	15 (3)	2583.2	5.8	33.1 (7.5–67.8)	53.9 (22.3–87.5)	77.6 (49.9–93.7)	68.9 (41.3–90.0)	98.4 (91.9–99.9)	2600 (<0.001)
Hip fracture during follow-up									
From 50 years	189 (8)	66128.1	2.9	12.1 (6.0–21.2)	4.8 (1.1–10.8)	4.3 (0.9–10.2)	13.8 (7.5–22.8)	26.6 (17.1–37.4)	59.1 (0.003)
From 60 years	150 (8)	37201.8	4.0	12.5 (5.6–21.9)	9.7 (4.0–18.1)	9.9 (3.9–19.0)	11.1 (5.6–18.9)	34.5 (23.6–46.8)	168.5 (<0.001)
From 71 years	100 (8)	15239.4	6.6	28.0 (16.2–43.4)	9.0 (3.1–19.6)	9.7 (3.3–20.2)	19.7 (10.3–33.2)	54.5 (41.1–68.7)	63.3 (<0.001)
From 82 years^i^	30 (6)	2569.7	11.7	57.0 (29.8–81.9)	34.9 (15.9–60.0)	28.4 (10.0–57.6)	27.8 (9.0–58.1)	86.7 (67.3–97.0)	154.8 (<0.001)
Hip fracture within 10 years									
From 50 years	9 (0.4)	22623.6	0.4	Not analyzed	Not analyzed	Not analyzed	Not analyzed	Not analyzed	Not analyzed
From 60 years	22 (1)	17341.4	1.3	60.5 (27.8–87.6)	46.9 (19.8–76.1)	39.6 (16.5–69.4)	53.0 (27.4–85.0)	92.2 (71.8–99.3)	124.7 (0.013)
From 71 years	41 (3)	10792.8	3.8	40.7 (17.6–68.2)	24.8 (8.7–47.3)	14.9 (3.7–37.5)	42.1 (22.7–64.4)	81.7 (66.2–93.1)	117.4 (0.001)
From 82 years^i^	30 (6)	2569.7	11.7	57.0 (29.8–81.9)	34.9 (15.9–60.0)	28.4 (10.0–57.6)	27.8 (9.0–58.1)	86.7 (67.3–97.0)	154.8 (<0.001)

ATC = Anatomical Therapeutic Chemical classification system; PYAR = person-years at risk; rIDI = relative integrated discrimination improvement; V_FRAX_ = FRAX variables.

a rIDI measures improved prediction when comparing the full model with V_FRAX_.

b V_FRAX_ includes: age, height, weight, current smoking, high alcohol consumption, previous fracture, secondary osteoporosis, rheumatoid arthritis, parent fractured hip, and glucocorticoid use.

c The comorbidity model includes: number of comorbidities, diabetes mellitus type 2, cardiovascular disease, cancer, and other comorbidities (including secondary osteoporosis and rheumatoid arthritis).

d The medication model includes medication grouped into the major ATC groups.

e The behavioral model includes current and former smoking, alcohol consumption, leisure-time and work physical activity, civil status, educational level, and impaired cognitive function.

f The full model is a combination of ^b–e^ and includes all variables in V_FRAX_, plus comorbidity (number of comorbidities, diabetes, cardiovascular disease, cancer, other), medication use (ATC groups) and behavioral factors (former smoking, leisure-time and work physical activity, civil status, educational level, and impaired cognitive function).

g Duplicate information, follow-up time from 82 years is maximum 6.7 years, median 5.2 years.

h Follow-up time from 82 years is maximum 6.7 years, median 5.4 years.

i Duplicate information, follow-up time from 82 years is maximum 6.7 years, median 5.3 years.

**Figure 4:**
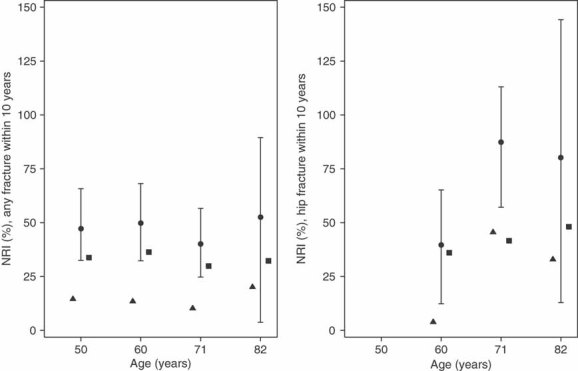
Net reclassification improvement (NRI). NRI for prediction of any fracture (left panel) and hip fracture (right panel) when comparing the full model with FRAX variables (V_FRAX_). Triangles (▴) represent NRI among events, squares (▪) NRI among nonevents, and circles (•) the combined NRI with 95% confidence interval error bars.

The prognostic value of V_FRAX_ estimated as R^2^ was at best 17% for any fracture (Table 2). Comorbidity and medication were rare at age 50 years and the models had low predictive ability, whereas the behavioral model's performance was twice that of V_FRAX_. At ages 60 to 82 years, the four separate models had more similar prognostic values. The full model explained 25% to 45% of the variation in time to any fracture occurring within 10 years. Further, the full model could discriminate risk for any fracture better than V_FRAX_ with rIDI values between 164% and 243% (Table 2). The NRI showed around 50% improved classification when using the full model compared to V_FRAX_, at all ages (Fig. 4). The improvement was largest among nonevents. Harrell's C for V_FRAX_ was 0.64 (0.60–0.68) at age 50 years, 0.62 (0.57–0.66) at age 60 years, 0.59 (0.55–0.63) at age 71 years, and 0.66 (0.61–0.72) at age 82 years. The corresponding C coefficients for the full model were 0.71 (0.68–0.75), 0.72 (0.67–0.75), 0.67 (0.63–0.70), and 0.66 (0.71–0.80), respectively.

For hip fractures, the prognostic value of V_FRAX_ was 41% to 60%. Also, the comorbidity, medication, and behavioral models had higher R^2^ values compared to those for any fracture. The full model explained at least 80% of the variability in time to hip fracture. Discrimination also improved, with rIDI varying between 83% and 124% (Table 2) and NRI varying between 40% and 87% (Fig. 4). The NRI among events was low (4%) at age 60 years, but at older ages it was more similar to the NRI among nonevents. Harrell's C for V_FRAX_ was 0.77 (0.68–0.85) at age 60 years, 0.71 (0.63–0.77) at age 71 years, and 0.79 (0.71–0.86) at age 82 years. Corresponding C coefficients for the full model were 0.87 (0.79–0.94), 0.84 (0.80–0.89), and 0.88 (0.82–0.93), respectively.

The prognostic values for time to a second fracture within 10 years with V_FRAX_ ranged between 14% and 33% whereas the full model had R^2^ values ranging between 61% and 98%. The R^2^ values of medication increased with increasing age.

BMD at the femoral neck was measured in 461 men (88%) at age 82 years. V_FRAX_ with the addition of BMD explained 34.2% (15.7% to 57.0%) of all fractures (*n* = 55) and 58.7% (29.8% to 85.4%) of hip fractures (*n* = 25). Corresponding R^2^ values for the full model also including BMD were 62.4% (42.8% to 80.2%) and 96.8% (87.3% to 99.6%), respectively.

The performances of V_FRAX_ and the full model were also investigated from a screening perspective. Based on each model, the highest quintile of predicted hazard was compared with the lowest. The rate contrasts between the lowest and highest quintile of predicted fracture risk were more pronounced with the full model compared with V_FRAX_. The sensitivity and the odds of suffering a fracture and being in the highest risk quintile (OAPR) were calculated (Table 3). The sensitivity was generally modest. Nevertheless, a high proportion of men within the highest quintile of predicted risk with the full model will suffer a fracture. For instance, one-third at age 71 years and two-thirds at age 82 years, of those predicted as high risk for any fracture also suffered a fracture (OAPR 1:3.1 and 1:1.6, respectively). The corresponding odds of hip fracture were one-fifth and one-third.

**Table 3:** Comparison of V_FRAX_ and the Full Model

	V_FRAX_^a^					Full Model^b^				
		
	Rate/1000 PYAR Q1	Rate/1000 PYAR Q5	Hazard ratio (95% CI) Q5 versus Q1^c^	Sensitivity (%)	OAPR^d^	Rate/1000 PYAR Q1	Rate/1000 PYAR Q5	Hazard ratio (95% CI) Q5 versus Q1^c^	Sensitivity (%)	OAPR^d^
Any fracture within 10 years										
From 50 years	2.4	9.6	3.99 (2.05–7.76)	22	1:7.7	1.8	13.0	7.44 (3.54–15.64)	28	1:5.8
From 60 years	4.1	10.9	2.67 (1.43–4.96)	18	1:8.3	3.7	15.9	4.27 (2.31–7.86)	22	1:6.6
From 71 years	8.5	22.5	2.67 (1.54–4.62)	18	1:3.6	7.6	25.7	3.46 (1.99–6.01)	20	1:3.1
From 82 years^e^	12.8	44.3	3.43 (1.44–8.17)	20	1:2.4	7.4	58.0	7.74 (2.69–22.25)	29	1:1.6
Two fractures within 10 years										
From 50 years	0.66	3.5	5.34 (1.55–18.46)	25	1:31	0.44	4.2	9.54 (2.21–41.1)	31	1:24
From 60 years	2.3	3.7	1.57 (0.64–3.83)	13	1:54	0.59	6.7	11.34 (2.66–48.38)	34	1:21
From 71 years	2.4	6.9	2.87 (1.03–7.96)	18	1:15	0.94	14.0	14.95 (3.56–62.89)	37	1:7.2
From 82 years^e^	4.2	6.3	1.61 (0.27–9.64)	13	1:19	0.00	25.8	2.34 · × 10^10^ (6.6 · × 10^9^–8.3 · × 10^10^)	100^f^	1:7.8^f^
Hip fracture within 10 years										
From 50 years	na	na	na	na	na	na	na	na	na	na
From 60 years	0.57	3.9	6.80 (1.53–30.12)	27	1:28	0.28	4.2	15.04 (1.98–114.4)	37	1:20
From 71 years	1.9	9.3	5.02 (1.71–14.76)	24	1:11	0.43	13.1	33.04 (4.48–243.9)	48	1:5.3
From 82 years^e^	3.8	36.3	9.58 (2.20–41.71)	31	1:3.7	1.9	41.0	22.29 (2.97–167.1)	42	1:2.8

ATC = Anatomical Therapeutic Chemical classification system; na = not analyzed; OAPR = odds of being affected given a positive result; PYAR = person-years at risk; Q = quintile; V_FRAX_ = FRAX variables.

a V_FRAX_ includes: age, height, weight, current smoker, high alcohol consumption, previous fracture, secondary osteoporosis, rheumatoid arthritis, parent fractured hip, and glucocorticoid use.

b The full model includes all variables in V_FRAX_ and: comorbidity (number of comorbidities, diabetes, cardiovascular disease, cancer, other), medication use (ATC groups), behavioral factors (former smoker, leisure-time and work physical activity, civil status, educational level, and impaired cognitive function).

c Hazard ratio for highest (Q5) versus lowest (Q1) quintile of predicted fracture rate, sensitivity of prediction, and the odds of having a fracture for men in the highest quintile at different ages based on the two models.

d Odds of having a fracture while being in highest 20% of predicted risk.^35^

e Rate is for 6.7 years of follow-up.

f Calculated manually since the calculation tool^35^ reached its limit.

We further investigated whether discrimination, estimated by rIDI, could be improved by addition of other exposures with potential influence on fracture risk. Addition of vitamin D and retinol concentrations and the number of previous falls to the full model improved discrimination of any fracture by 29% (*p* = 0.01) at age 71 years and by 62% (*p* = 0.002) at age 82 years. Hip fracture discrimination was improved by 15% (*p* = 0.02) and 27% (*p* = 0.05), and two fractures by 16% (*p* = 0.01) and 110% (*p* < 0.001), respectively. The number of previous falls, retrieved from the NPR, contributed a small part of this added discrimination, except at age 82 years, where the number of previous falls added 43% (*p* = 0.03) discrimination of any fracture to the full model and 110% (*p* = 0.03) to V_FRAX_. Self-reported number of falls in the previous year did not improve discrimination at age 71 years (data not shown).

## Discussion

The present population-based study of men followed from their 50s until old age shows that the addition of comorbidity, medication, and behavioral factors to the clinical components of FRAX can substantially improve the ability to identify men at high risk of fracture, especially hip fracture and a second fracture of any type. Furthermore, using information on comorbidity, medicine use, and behaviors, we can better identify those who will not suffer a fracture. The overall enhanced classification is illustrated by a net reclassification improvement between 40% and 87%, and a larger proportion of men actually suffering a fracture when predicted to be in the highest risk category.

The FRAX algorithm was developed to easily identify individuals at high 10-year risk of fracture likely to benefit from pharmaceutical treatment to improve bone density.^20^ Other interventions than pharmaceutical treatment need to be considered^12^ because a majority of fractures occur in persons without osteoporosis.^12^ Physical exercise^40^ and review of medications with the purpose of reducing dosage or completely withdrawing fall-risk–increasing drugs^41^, ^42^ can reduce the fall rate and possibly the fracture rate. Moreover, the injury impact on the hip by a fall can be reduced by hip protectors if they are made available to frail, older people.^15^, ^43^

Validation of FRAX in men has been scarce.^20^ This is in part because the beta coefficients for variables in FRAX have not been published. In a retrospective case-control analysis from Australia including 144 women and 56 men aged 60 to 90 years, FRAX with BMD was a poor predictor of fragility fracture in men but was more accurate in women.^5^ Although this discrepancy may have been due to chance, it has been argued that hip fracture–related comorbidity is a larger problem in men than in women.^16^, ^44^ Recent studies from the United States^45^ and United Kingdom^46^, ^47^ also indicate that comorbidity information can improve fracture prediction when added to the FRAX score. Our results imply that modeling variables in FRAX (V_FRAX_) in a population-based setting of men has a limited discriminative capacity to identify those who will suffer a fracture in the future, although the estimates for hip fractures seem better than for any fracture, especially if BMD information is available. We were not able to use the FRAX algorithm to calculate the FRAX score. The predictive capacity of the FRAX variables (V_FRAX_) was, however, similar to that shown by others using the FRAX algorithm.^46^, ^48^ Further, the actual FRAX algorithm may have performed even worse in our setting because it was developed as an average score of several cohorts at different settings. Additionally, we cannot tell how the other variables included in our analysis would perform if added to the FRAX score itself.

One criticism of the FRAX algorithm is that it does not include history of falls.^49^ Fall history did not contribute markedly to our full model at age 82 years, perhaps because fall history may be related to factors already included in the model,^20^, ^50^ or because is a too crude^50^ or poor measure of impaired balance, or both. The notion that fall history seemed to be of more importance among the oldest age group is in line with previous research findings of attenuated importance of BMD in osteoporotic fracture risk prediction with increasing age.^12^, ^51^

Because the risk of a subsequent fracture is highest in the year after a first fracture, it has been suggested that identification of those at high risk for recurrent fractures should be prioritized and that the treatment should have short-term effects in order to reduce the risk of a subsequent fracture.^52^ Our full model showed high prognostic value for two consecutive fracture events.

The strengths of our study include the population-based cohort of men with similar age, the high participation rate, long follow-up, and extensive repeated investigations, and linkage with official registers for the complete identification of all fractures, comorbid conditions, and previous falls. We were also able to assess the influence of competing risk from mortality.

Our study also has limitations. We did not use a validation dataset. To limit overly optimistic estimates, R^2^, NRI, and Harrell's C were calculated as the medians of 1000 bootstrap samples.^38^ Self-reported medications may be underreported by persons with polypharmacy or with a higher degree of cognitive impairment, leading to attenuated influence of medication on our estimates. BMD was only measured at the age 82 years investigation. However, a recent study demonstrates that more than 80% of patients with a FRAX designation of “high risk” of any fracture, calculated without BMD, also had osteoporosis.^53^ Still, around 80% of all fractures in a similar setting did not have osteoporosis.^7^ Our cohort is relatively small, with a limited number of fractures, especially after age 82 years, which means that there is a risk of overfitting using our multivariable models. However, the results point in the same direction for all ages and outcomes, and taking the number of covariates into account^33^ did not change our conclusions. Finally, inclusion of only Swedish men may limit generalization to women and other populations.

Prediction deals with several issues: determination of variables that contribute to the explanation of variation in time to event, predictive accuracy, and classification of individuals for clinical decisions.^54^ The first of these issues was investigated by the prognostic (R^2^) values that were developed for time to event data.^33^ The models' added discriminative ability was investigated by the rIDI, and the improvement of risk classification was measured as NRI. Although these two measures are suggested to be more sensitive estimates of discrimination and reclassification than the C statistic,^34^, ^36^ we could see improved discrimination with the full model also using the C statistic. In a recent reanalysis of data, FRAX, compared to age and BMD alone, improved the classification of fractures.^55^ This improvement was not observed using the C statistic.^4^ The screening performance^35^ partly investigates individual classification. Although we can demonstrate moderate to high prognostic values and improved discrimination and reclassification with our full model, sensitivity (discrimination at the individual level) was modest, a common feature of prediction models.^56^

Some of the components of FRAX are naturally also included in our other models. For instance, glucocorticoid use was included in our medication model, rheumatoid arthritis and the other diseases in V_FRAX_ were included in our diseases model, and current smoking and alcohol consumption were included in our behavioral model., To avoid collinearity, these components were not added twice when evaluating the full model or the nested models. Therefore, it is essential to emphasize that each group of variables added to V_FRAX_ contributed to the predictive ability of the full model, indicating that all categories are important.

One may argue that many comorbidities are known to increase fracture risk and that they could be entered into the FRAX tool as secondary causes of osteoporosis. Several of the diseases in our comorbidity model were tested within the FRAX cohorts in the development of FRAX, but it was concluded that “there was no evidence that these risk variables had any significant importance for fracture” (eg, stroke: hazard ratio [HR] for hip fracture, unadjusted for BMD; 1.20; 95% CI, 0.78–1.84).^57^ However, the lack of association might well be explained by a self-selection phenomenon: frail individuals are less like likely to be included in a cohort study. Yet two important strengths of the FRAX tool are its availability and that it is easy to use for clinicians without expert knowledge in the field of osteoporosis. We have therefore used as secondary causes of osteoporosis those that are suggested in the FRAX online tool. It should be emphasized that the information used in our full model can be retrieved from a patient's medical record or by asking the patient. The ease with which this is done will differ between settings. Moreover, compilation of clinical risk factors will in the future also become more easily accessible with the development of electronic patient records.^58^ A cognitive function test takes only a few minutes to complete. Our results suggest that bone scans and serum vitamin analyses could add further discriminatory capacity. Future studies can investigate whether these markers can guide the physician to whether the primary treatment of a person at high risk should be focused toward improving bone density or balance, or both.

Although we do not provide a ready tool for use by physicians in their daily work, our results highlight the importance of increased awareness of risk factors for fractures. It also emphasizes that future prediction tools aiming at identifying people at high risk of fracture should include more information on comorbidity, medication use, and behavioral factors than what is currently included in the FRAX tool.

We conclude that fracture prediction in older men can be considerably improved by the addition of easily accessible clinical and behavioral risk factors to the variables included in the FRAX algorithm. The full model was especially powerful for identifying elderly men at high risk of hip fractures and those with high risk of two fractures.
