# Supplementary material for: Prediction of fracture risk in men: A cohort study
Source: J Bone Miner Res. 2011 Dec 20;27(4):797–807. doi: 10.1002/jbmr.1498 (PMC3415621; doi:10.1002/jbmr.1498)
Supplement: Supplementary file 1 [file jbmr0027-0797-SD1.doc]

**eTable 1. Definitions of comorbidities** and comorbidity prevalence for each baseline age presented as numbers (percentages)

| **Disease** | **ICD-10 codes** | **ICD-9 codes** | **ICD-8 codes** | **Age 50 (n=2322)** | **Age 60 (n=1852)** | **Age 71 (n=1221)** | **Age 82 (n=526)** |
| --- | --- | --- | --- | --- | --- | --- | --- |
| Rheumatoid arthritis | M05 | 714A, 714B, 714C, 714W | 712,10, 712,38 | 0 (0) | 0 (0) | 3 (0.2) | 4 (0.8) |
| ***Secondary osteoporosis*** |  |  |  | 19 (0.8) | 21 (1) | 19 (2) | 11 (2) |
| Liver disease ^a^ | B150, B16-B18, I85, K70-K77 | 070A, 070C-070X, 456A, 456B, 570-573, V42H | 070,01; 070,03; 070,04; 070,07; 070,09; 456,00; 570-573 | 5 (0.2) | 7 (0.4) | 9 (0.7) | 1 (0.2) |
| Type 1 diabetes mellitus ^b^ |  |  |  | 13 (0.6) | 11 (0.6) | 7 (0.6) | 8 (2) |
| Hypogonadism | E230, E291 | 257C | 757,10 | 0 (0) | 0 (0) | 0 (0) | 0 (0) |
| Malnutrition | E40-E46, R634 | 260-263, 783C | 269,10, 788,40 | 0 (0) | 0 (0) | 1 (0.08) | 1 (0.2) |
| Thyreotoxicosis | E05 | 242 | 242 | 1 (0.04) | 3 (0.2) | 3 (0.2) | 5 (1) |
| ***Cardiovascular diseases*** |  |  |  | 35 (2) | 217 (12) | 316 (26) | 263 (50) |
| Congestive heart failure | I090, I110, I13, I42-I43, I50-I51 | 393A, 398, 402A, 402X, 404A, 404B, 404X, 425, 428, 429 | 398,99; 393,99; 402,99; 404,99; 425; 427,0; 427,1; 428,99; 429,99 | 3 (0.1) | 12 (0.6) | 36 (3) | 51 (10) |
| Ischemic heart disease | I20-I25 | 410-414 | 410-414 | 10 (0.4) | 99 (5) | 163 (13) | 135 (26) |
| Valvular disease | I050-I089, I091, I33-I39 | 394-397, 421, 424 | 394-397, 421, 424 | 1 (0.04) | 4 (0.2) | 17 (1) | 21 (4) |
| Cardiac arrythmias | I44-I49, R00 | 426-427 | 427,2; 427,9; 782,20 | 0 (0) | 0 (0) | 54 (4) | 87 (16) |
| Pulmonary circulation disorders | I26-I28 | 415-417 | 426 | 0 (0) | 0 (0) | 4 (0.3) | 10 (2) |
| Hypertension | I109, I119, I12, I15 | 401-405 | 400-404 | 17 (0.7) | 77 (4) | 98 (8) | 98 (19) |
| Cerebrovascular disease | I60-I69 | 430-438 | 430-438 | 3 (0.1) | 31 (2) | 75 (6) | 61 (12) |
| Peripheral vascular disorders ^c^ | I70-I82, I85-I89, R029 | 440-447, 452-453, 456 | 440-447, 452, 453, 456 | 7 (0.3) | 40 (2) | 53 (4) | 37 (7) |
| ***Cancer*** |  |  |  | 6 (0.3) | 33 (2) | 75 (6) | 85 (16) |
| Haematological malignancies | C81-C96, D477-D479 | 200-208 | 200-207 | 0 (0) | 4 (0.2) | 7 (0.6) | 5 (1.0) |
| Solid tumour without metastases | C00-C76, Z510, Z511, Z926 | 140-195 | 140-195 | 6 (0.3) | 29 (2) | 71 (6) | 82 (16) |
| Metastatic cancer | C77-C80, C979 | 196-199 | 196-199 | 0 (0) | 1 (0.05) | 1 (0.08) | 1 (0.2) |
| ***Other diseases*** |  |  |  | 140 (6) | 532 (29) | 589 (48) | 352 (67) |
| Neurological disease ^d^ | G00-G99, F00-F09, R25-R29, R298, R296, R40, R41, R47, R48 | 320-359, 290, 293-294, 310, 784D, 784F, 780A, 781C, 781D | 320-358; 290; 293; 294; 309; 780,0; 780,4; 781,51; 781,52; 781,59 | 8 (0.3) | 35 (2) | 50 (4) | 54 (10) |
| **Disease** | **ICD-10 codes** | **ICD-9 codes** | **ICD-8 codes** | **Age 50 (n=2322)** | **Age 60 (n=1852)** | **Age 71 (n=1221)** | **Age 82 (n=526)** |
| Chronic pulmonary disease | D860, D862, E662, E840, E849, J40-J67, J684, J701, J703, J84, J850, J953, J961, J969, J98, J99 | 490-505, 506E, 506X, 508B, 515-517, 519B, 519D, 519E, 519W | 490-493; 515-517; 518,99; 519,92 -519,94 | 3 (0.1) | 19 (1) | 25 (2) | 32 (6) |
| Infectious disease ^e^ | A00-B09, B159, B25-B99, J00-J32, J851-J869, M00-M01, M726, N300, N390, T880 | 001-066, 070B, 071-078, 079A-079D, 079W, 079X, 080-139, 460-466, 473, 480-491, 510-511, 519C, 590, 595, 597, 998F, 999D, 790H, 790W | 000-068; 070,02; 071-136; 460-466; 470-474; 80-486; 503; 510; 511; 519,91; 590; 595; 597; 998,50; 999,20; 999,30 | 15 (0.6) | 97 (5) | 137 (11) | 121 (23) |
| Diabetes ^f^ | E10-E14 | 250 | 250 | 6 (0.3) | 37 (2) | 44 (4) | 39 (7) |
| Endocrine disorders excluding diabetes | E00-E07, E15-E35, E89 | 240-246, 251-259 | 240-246, 251-258 | 9 (0.4) | 33 (2) | 33 (3) | 21 (4) |
| Renal disease | N00-N12, N14-N19, R34, R392, Z992 | 403, 404, 580-589, V42A, V45B, V56 | 403; 404; 580-584; 590,1; 593,1-593,2; 753,02; Y290,1 | 2 (0.09) | 6 (0.3) | 7 (0.6) | 16 (3) |
| Immune deficiencies, including HIV | B20-B24, D80-D84, D89 | 279, 079J, V02J | 275,00; 275,10; 275,9 | 0 (0) | 0 (0) | 0 (0) | 0 (0) |
| Other (non-cancer) haematological diseases | D69-D77, D45-D46, D471-D473 | 288-289 | 288-289 | 0 (0) | 3 (0.2) | 2 (0.2) | 4 (0.8) |
| Coagulopathy | D65-D68 | 286-287 | 286-287 | 0 (0) | 1 (0.05) | 1 (0.08) | 2 (0.4) |
| Obesity | E65-E66 | 278 | 277,99 | 0 (0) | 0 (0) | 10 (0.8) | 3 (0.6) |
| Nutritional deficiencies | E40-E64, E90, R634 | 260-269, 783C | 260-269; 788,40 | 0 (0) | 0 (0) | 1 (0.08) | 2 (0.4) |
| Fluid and electrolyte disorders | R631, E86-E87 | 276 | 788,00; 788,10; 788,19; 788,69; 788,70; 788,61; 788,92 | 0 (0) | 0 (0) | 0 (0) | 1 (0.2) |
| Blood loss anaemia | D629 | 285B | 280,00 | 0 (0) | 0 (0) | 0 (0) | 0 (0) |
| Deficiency and other anemias | D50-D61, D64 | 280-284, 285A, 285W, 285X | 280,08; 280,09; 281-285 | 4 (0.2) | 4 (0.2) | 10 (0.8) | 15 (3) |
| Alcohol abuse ^g^ | F10, K70, K852, R780, Z502, Z721, | 291, 303, 305A, 790D | 261,00; 262,00; 291; 303; 571,00; 571,01; 980 | 9 (0.4) | 44 (2) | 26 (2) | 5 (1) |
| Drug abuse | F11-F19, R781-R786, Z503, Z722 | 292, 304, 305B, 305X | 294,30; 304; 971 | 0 (0) | 1 (0.05) | 1 (0.08) | 0 (0) |
| Psychoses | F20-F29, R44 | 295, 297-299, 780B | 295; 297-299; 780,10 | 0 (0) | 10 (0.5) | 5 (0.4) | 0 (0) |
| **Disease** | **ICD-10 codes** | **ICD-9 codes** | **ICD-8 codes** | **Age 50 (n=2322)** | **Age 60 (n=1852)** | **Age 71 (n=1221)** | **Age 82 (n=526)** |
| Affective disorders | F30-F39 | 296, 311 | 296 | 4 (0.2) | 44 (2) | 30 (2) | 12 (2) |
| Other psychiatric disease | F40-F99, Z504 | 300-302, 307-309, 312-319 | 300-302, 305-315 | 0 (0) | 0 (0) | 2 (0.2) | 0 (0) |
| Transplantation | Z940-Z944, T86 | V42, 996W | 997,70 | 0 (0) | 0 (0) | 1 (0.08) | 0 (0) |
| Rheumatoid arthritis and collagenosis | M05-M09, M30-M35 | 279N, 710, 713D, 713H, 714, 720, 725, 729A-729E | 712; 714; 717,98; 718; 734 | 5 (0.2) | 20 (1) | 20 (2) | 15 (3) |
| Other diseases of bone and muscle ^h^ | M12-M25, M40-M83, M858-M859, M86-M90, M99 | 715-738 | 713; 714,0; 714,9; 715-738 | 29 (1) | 154 (8) | 189 (15) | 129 (24) |
| Injury ^i^ | S00-T35 (Excluding S12, S22, S32, S42, S52, S62, S72, S82, S92), T66-T79, T90-T98 | 801-804, 830-959, 990-994, 995F | 801-804, 830-959, 990-994 | 19 (0.8) | 74 (4) | 97 (8) | 66 (12) |
| Intoxication | T36-T65 | 960-989 | 960-989 | 3 (0.1) | 10 (0.5) | 11 (0.9) | 6 (1) |
| Diseases of the eye | H00-H59 | 390-379 | 360-379 | 10 (0.4) | 58 (3) | 77 (6) | 42 (8) |
| Diseases of the ear | H60-H95 | 380-389 | 380-389 | 9 (0.4) | 27 (1) | 38 (3) | 21 (4) |
| Stomach and bowel diseases ^j^ | K20-K31, K50-K67 | 530-537, 555-569 | 530-537, 560-569 | 28 (1) | 95 (5) | 116 (10) | 66 (12) |
| Osteoporosis ^k^ | M80-M82 | 733A | 723,00 | 0 (0) | 0 (0) | 0 (0) | 3 (0.6) |
| Type 2 diabetes mellitus ^l^ |  |  |  | 30 (1) | 103 (6) | 182 (15) | 94 (18) |
| ***Number of previous falls*** | W01-W19 | E880-E888 | E880-E887 |  |  |  |  |
| 1, No. (%) |  |  |  | 8 (0.3) | 40 (2.2) | 69 (5.6) | 53 (10.1) |
| 2, No. (%) |  |  |  |  | 10 (0.5) | 15 (1.2) | 16 (3.0) |
| 3 or more, No. (%) |  |  |  |  | 4 (0.2) | 7 (0.6) | 5 (1.0) |

^a^ Includes hepatitis but not hepatitis A without hepatic coma

^b^ Defined as men with insulin treatment at age 50 or 60 years, excluding men using oral antidiabetics

^c^ Excludes lower extremity varices and haemorrhoids

^d^ Includes organic dementia/delirium etc

^e^ Includes hepatitis A without hepatic coma. Other hepatitis classified under liver disease. HIV-related problems classified under immunodeficiency.

^f^ No split into uncomplicated/complicated

^g^ Includes increased alcohol level in blood

^h^ Includes osteoporosis

^i^ Includes late complications but excludes complications to medical treatment and intoxications. Excludes fractures but includes fractures of the scull.

^j^ Excludes hernia and diseases of the appendix

^k^ Not included in statistical models since all men with osteoporosis had also suffered a previous fracture

^l^ Determined at the clinical investigations according to guidelines current at that time and included as a covariate in the comorbidity model and in the full model.

**eTable 2. Prevalence of medications by ATC code at the different baseline ages^a^**

| **ATC-group** | **Age 50 (n=2322)** | **Age 60 (n=1852)** | **Age 71 (n=1221)** | **Age 82 (n=526)** |
| --- | --- | --- | --- | --- |
| A | 23 (1) | 128 (7) | 262 (22) | 149 (28) |
| B | 6 (0.3) | 36 (2) | 275 (22) | 266 (51) |
| C | 129 (6) | 493 (27) | 454 (37) | 319 (61) |
| D | 0 | 6 (0.3) | 22 (2) | 11 (2) |
| G | 0 | 5 (0.3) | 26 (2) | 63 (12) |
| H | 7 (0.3) | 20 (1) | 34 (3) | 39 (7) |
| J | 0 | 4 (0.2) | 7 (0.6) | 9 (2) |
| L | 0 | 1 (0.05) | 9 (0.7) | 20 (4) |
| M | 0 | 27 (2) | 70 (6) | 68 (13) |
| N | 53 (2) | 135 (7) | 315 (26) | 132 (25) |
| P | 0 | 2 (0.1) | 8 (0.7) | 1 (0.2) |
| R | 4 (0.2) | 53 (3) | 111 (9) | 50 (10) |
| S | 0 | 10 (0.5) | 27 (2) | 44 (8) |
| V | 0 | 0 | 1 (0.1) | 0 |

^a^ Values are numbers (percentages)
